# The Destructive Capacity of Drug Abuse: An Overview Exploring the Harmful Potential of Drug Abuse Both to the Individual and to Society

**DOI:** 10.1155/2013/450348

**Published:** 2013-07-16

**Authors:** Thomas Peter Fox, Govind Oliver, Sophie Marie Ellis

**Affiliations:** King's College Hospital London, NHS Foundation Trust, London SE5 9RS, UK

## Abstract

From a public health perspective, substance abuse has long been a source of major concern, both for the individual's health and for wider society as a whole. The UK has the highest rates of recorded illegal drug misuse in the western world. In particular, it has comparatively high rates of heroin and crack cocaine use. Substances that are considered harmful are strictly regulated according to a classification system that takes into account the harms and risks of taking each drug (see the tables) (Nutt et al. (2007)). The adverse effects of drug abuse can be thought of in three parts that together determine the overall harm in taking it: (1) the direct physical harm of the substance to the individual user, (2) the tendency of the drug to induce dependence, and (3) the effect of abuse of the drug on families, communities, and society (Gable (2004, 1993)). In this report, we discuss published evidence relating to the harm of substance misuse and consider the neuropsychopharmacological mechanisms behind addiction in an attempt to gain an improved picture of the potential devastation that abuse of these substances may evoke.

## 1. The Direct Physical Harm of the Substance to the Individual User 

The physical harm caused by a drug needs to be considered in terms of its acute toxicity, as well as its likelihood to produce long-term health problems.

### 1.1. Acute Toxicity

Acute toxicity is the adverse effect produced by a drug following either a single exposure or multiple exposures under 24 hours apart. It is assessed by measuring the ratio of lethal dose to therapeutic dose. Research conducted on 20 human and nonhuman lethal doses of abused substances, which are distributed widely in Europe and North America, identified intravenous heroin to have the greatest direct physiological toxicity. Hallucinogens in general appeared to have the least [1]. The clinical sequelae of exposure to toxic levels of a drug can be present in a specific set of symptoms referred to as toxidrome. Cocaine, for example, at low doses produces euphoria, reduced fatigue, and a perception of increased mental acuity. Higher doses may result in several undesirable side effects including irritability, paranoia, panic, repetitive stereotyped behaviour, diaphoresis, mydriasis, tachyarrhythmias, stroke, and seizure [2, 3]. A thorough exploration of common and important toxidromes is beyond the scope of this paper but detailed information for UK healthcare professionals can be found on the clinical toxicology database [4].

### 1.2. Long-Term Health Problems

As well as acute physical harm, many drugs when used repeatedly over time have chronic physical consequences. The long-term health problem can either be directly related to the effect of the drug or due to the method of drug administration. 

Several drugs including marijuana are ingested by smoking and put abusers at increased risk of chronic cough, bronchitis, and lung and upper airway cancers. The long-term adverse effects of cigarette smoking are such that they reduce life expectancy by an average of ten years [6]. Drugs taken intravenously can lead to complications related to this route of delivery. These include thrombosed veins, bacterial endocarditis, abscesses, pneumonia, and liver disease [7]. In addition to the effects of the drug itself, street heroin may have additives that do not fully dissolve and result in further damage to blood vessels. Intravenous drug use and sharing of hypodermic needles open a gateway for potentially lethal blood-borne infections such as HIV and hepatitis. Statistics have shown that 21% of intravenous drug users are hepatitis B positive while 50% are hepatitis C positive [7]. In 2005, 28% of intravenous drug users reported directly sharing needles and syringes, which accounted for 5.6% of HIV diagnoses reported in the UK [8]. Heroin use carries a particularly significant mortality risk. Users are at twelve times greater risk of mortality than the general population and intravenous users, further 22 times greater risk, relative to noninjecting peers [9]. Chronic intranasal usage is also associated with specific problems such as degradation of the nasal septum [10]. A common but untrue belief is that the smoking of cocaine chemically breaks down tooth enamel and causes tooth decay. However, cocaine does often cause involuntary tooth grinding, known as bruxism, which can deteriorate tooth enamel and lead to gingivitis [11].

 Long-term health problems also result from the psychoneurobiological impact of chronic use. Chronic marijuana abuse has been shown to result in depression, anxiety, and, in some individuals with a predisposing vulnerability, schizophreniform disorder [13]. Hallucinogens can result in flashbacks and hallucinogen persisting perception disorder. Methylenedioxymethamphetamine (MDMA) and cocaine can both result in impulsiveness, irritability, sleep disturbance, anxiety, and addiction [14]. Many drugs of abuse also increase susceptibility and risk of suffering other conditions. Cocaine, for example, increases the risk of developing rare diseases such as systemic lupus erythematosus (SLE), vasculitis, Goodpasture's disease, Stevens-Johnson syndrome, and an array of kidney diseases [15–17]. It also doubles both the risks of hemorrhagic and ischemic strokes as well as increases the risk of myocardial infarction [18, 19].

## 2. The Tendency of the Drug to Induce Dependence

### 2.1. Drug Tolerance

To understand the nature of drug dependence it is first important to understand the concept of drug tolerance. Tolerance is commonly encountered with substance abuse and is described as a decrease in susceptibility to the effects of a given amount of drug as a result of previous exposure [4]. This means that increasingly larger doses are required to induce the desired effect. Whilst tolerance itself is not a particular problem to the health of the individual, it can have serious repercussions [21]. Firstly, there is usually little harm caused by most psychoactive drugs when taken in smaller quantities with sufficient time in between; however, once tolerance develops, larger and more frequent doses are administered that may be toxic and lead to direct harm from the substance itself. Secondly, tolerance to the different effects of a drug does not develop uniformly. This can be illustrated using alcohol; for example, tolerance often arises to the recreational effects of alcohol, but little tolerance develops to the lethal effects [22]. This results in a lowering of the therapeutic index. Finally, the mechanism that underlies tolerance contributes to the person's compulsion to take a drug resulting in socially unacceptable behaviour such as theft and fraud. This forms the basis of addiction [23].

### 2.2. Drug Dependence and Theories of Addiction

Dependence can be a physical or emotional adaptive state which results from the body's homeostatic response to repeated drug administration. Upon cessation of the drug, the homeostasis is lost and the dependence is unmasked, for example, cold turkey with heroin dependence [4]. The cessation of the drug and resulting chemical imbalance triggers negative emotions and behavioural disorder to be indicative of the disturbance. This is referred to as “reward deficiency syndrome.” Drug addiction may initially cause and then further proceed to exacerbate “reward deficiency syndrome” [24]. Another theory is the “drug for reward theory,” which states that addiction is the malfunctioning collision of both motivational systems (like versus want), stimulating pursuit of a substance that most probably no longer provides pleasure and in fact may be pathogenic [25].

### 2.3. Neurological Pathways in Addiction

**Figure 1:**
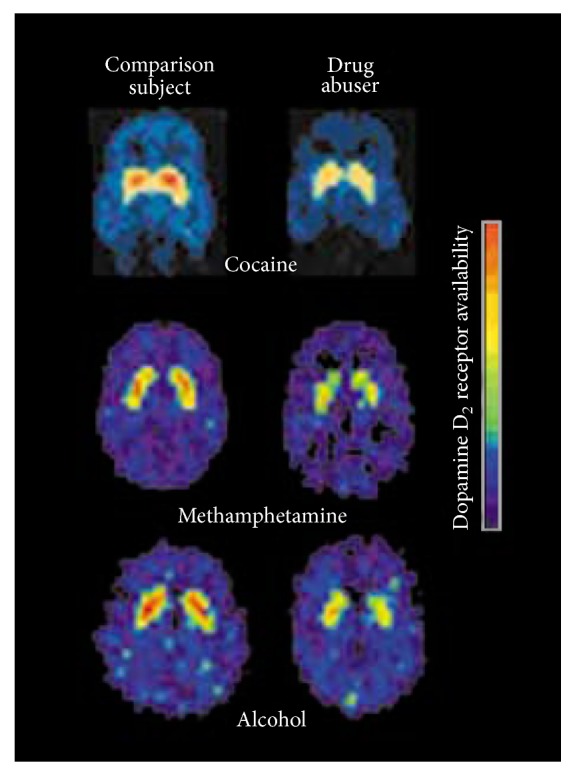
Decreased dopamine D2 receptor binding in drug users during withdrawal from cocaine, methamphetamine, and alcohol than in normal comparison subjects. Image from Carlson [5].

Reinforcers such as food and sex increase extracellular dopamine in the nucleus accumbens (NAcc) via the mesocorticolimbic pathway. This results in the sensation of reward. All known addictive drugs activate this pathway but at levels up to ten times greater than food [5]. As a result of the elevated levels of dopamine, compensatory changes take place that result in tolerance and dependence. This is due to long-term neuroadaptations in the dopaminergic system. There is an increase in transcription factors (DeltaFosB, CREB) as well as differential expression of proteins involved in synaptic plasticity [26]. There are also alterations to neurotransmission, and at a cellular level the morphology is altered by increased dendritic branching and spine density in the NAcc and prefrontal cortex (PFC) [26]. Another difference seen consistently in drug addicts is the decrease in dopamine D2 receptor binding in the NAcc (see Figure 1 [5]). This is because of the reducing number of dopamine receptors present if dopamine levels are repeatedly elevated. It is hypothesized that this dulling of the responsiveness of the brain's reward pathways contributes to the inability to feel pleasure, known as anhedonia, often observed in drug dependence [26, 27].

**Figure 2:**
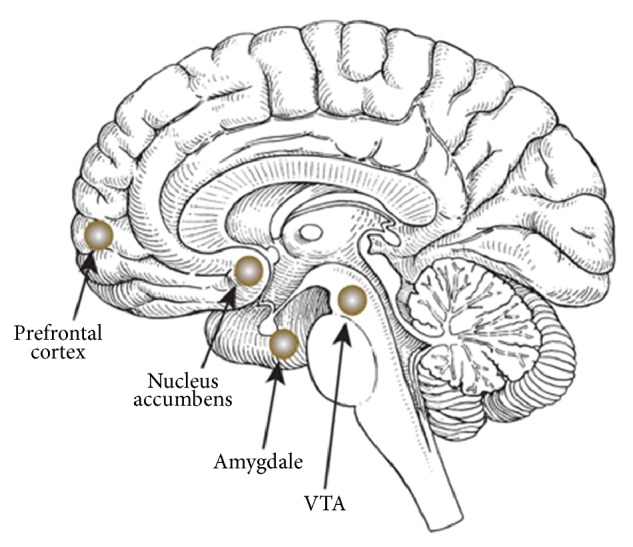
The brain centres involved in the mesocorticolimbic system [20].

Addiction comes about through an associative learning process characterized by compulsive drug taking, craving, and relapse [11, 21]. The pathway thought to be involved in addiction is the mesocorticolimbic pathway which consists of dopamine neurons projecting from ventral tegmental area (VTA) to the NAcc and prefrontal cortex (see Figure 2). As we have mentioned, this pathway plays a critical role in reward and reinforcement. Behaviours that activate this pathway tend to be repeated even if the experience was not considered pleasurable [28]. Most rewarding effects can be attenuated by blockade of dopamine in this region [29]. 

### 2.4. Physical Dependence to Opiates

**Figure 3:**
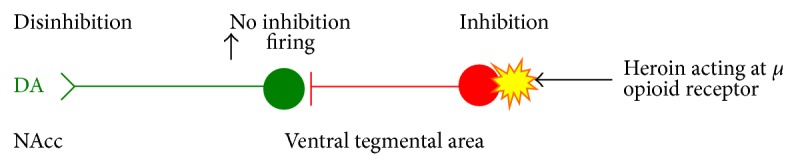
Heroin activates the inhibitory *μ* opioid receptor on the GABA neurons which results in an increase of dopamine release in the NAcc.

Heroin acts at endogenous opioid receptors in the VTA and NuAcc. Reward and reinforcement come about through disinhibition of the dopamine neurons in the VTA. These neurons are usually fire tonically but are inhibited by GABA interneurons. When heroin activates the *μ* opioid receptor on the GABA neurons, it inhibits them from firing thus removing the inhibition of the dopamine neurons. The result is an increase of dopamine release in the NAcc which is responsible for rewarding and reinforcing behaviour at that time (see Figure 3) [21].

Opioid receptors are found not only in the mesocorticolimbic system, but also in other systems such as the spinal cord and locus coeruleus (LC). The LC contains noradrenergic nuclei that are implicated in attention and arousal as well as the “fight or flight” autonomic response. Tolerance and dependence arise through chronic activation of opiate receptors, which leads to homeostatic compensatory changes. Acutely, heroin inhibits firing of LC neurons. With chronic use, the LC neurons return to their normal firing rates. This results in increasing doses of heroin being required to achieve the desired effect [21, 30].

Relapse is a very common problem during rehabilitation from opiate use [31, 32]. Associative cues develop by long-term potentiation during the drug use. Reexposure to the drug triggers these cues, making abstinence increasingly difficult and relapse likely [21]. Upon cessation of heroin, the user may suffer withdrawal symptoms which are essentially opposite to the acute effects and are considered an overshoot rebound to the initial drug-induced state (see Table 1) [12].

Whilst not life threatening, the symptoms are extremely unpleasant, often being flu-like in nature. The severity of withdrawal depends primarily on the intensity of the initial drug effects as well as the dose administered and frequency of use. The general health and personality of the patient are also thought to play a part [21].

### 2.5. Amphetamine and Cocaine

**Figure 4:**
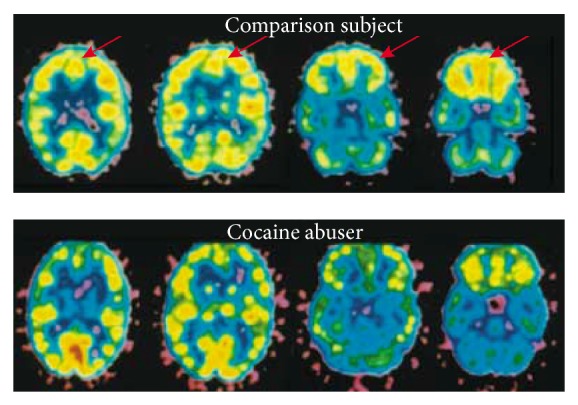
Lower relative glucose metabolism in the prefrontal cortex of a cocaine user than in a normal comparison subject. Image from Carlson [5].

Amphetamine is an indirect agonist of the catecholaminergic system. It causes release of catecholamines from the presynaptic endings as well as blocking the reuptake. It also inhibits monoamine oxidase activity and hence metabolism of catecholamines [26]. These stimulants act at the dopamine transporter (DAT) which is the main mechanism related to the reinforcing effects [21]. As with most drugs, tolerance is a problem as well as the reverse phenomenon known as sensitisation. This can occur spontaneously to some users on repeated intoxication with doses that previously only caused euphoria. If this happens, users may suffer acute behavioural changes that are virtually indistinguishable from paranoid schizophrenia [11]. After chronic use of cocaine, user's dopamine receptors become downregulated as they adapt to the constantly elevated levels of dopamine. Upon cessation, users suffer an increasingly bothersome withdrawal syndrome [33]. Positron emission tomography (PET) studies in chronic cocaine users have shown that there is reduced glucose metabolism in some brain areas when compared with nonusers (see Figure 4 [5]). It is hypothesised that the hypoactivity in these areas may underly some of the behaviors associated with cocaine addiction such as loss of motivation, impulsive drug taking, and the inability to abstain when exposed to drugs because of loss of inhibition [5, 34].

Another study which also used PET imaging showed evidence of drug cue-induced DA release in the amygdala and hippocampus. The preferential induction of DA release among cocaine users further suggested that these aspects of the limbic reward network might contribute to drug-seeking behavior [35].

A recent study investigating changes in drug use severity and physical health-related quality of life among untreated stimulant users concluded that the severity of cocaine use is linked directly to physical health-related quality of life; strong evidence of the drugs long-term health implications [36]. 

## 3. The Effect of Drug Use on Families, Communities, and Society

So far we have discussed the harm of addiction for the individual. However the implications of drug use extend far beyond the user, often damaging their relationships with their family, community, health workers, volunteers, and wider society [10]. 

### 3.1. Family and Children

One of the biggest impacts abuse has is upon the children or dependants of the abuser. The Hidden Harm report by the Advisory Council on the Misuse of Drugs estimated that there were between 250,000 and 350,000 children of problem drug misusers in the UK [37]. As with any drug, harmful effects on pregnancy cause much concern and cocaine is no exception with 90% of female users being of childbearing age [38]. The National Survey on Drug Use and Health (NSDUH) data collected during 2002 and 2003 indicates over 4% of pregnant women reporting using illicit drugs in the past month [39]. Prenatal cocaine use has immediate, short-term and long-term health implications. Immediate complications include increase of the risk of miscarriage and preterm labour (before 37 weeks of pregnancy). As a result, cocaine-exposed babies are more likely than unexposed babies to be born prematurely and with low birth weight. Premature and low-birth weight babies are at increased risk of health problems during the neonatal period, lasting disabilities such as learning difficulties, cerebral palsy, or even death [40, 41]. A recent study on school-aged children has demonstrated that prenatal cocaine exposure is reliably associated with impairments in attention and behavioural problems [42]. Children of individuals who use drugs are often abused or neglected as a direct result [43]. Studies have shown that the users need to find and fund their drug habit often taking precedence over the care of their children, leading to neglect [44]. These children may also lack proper immunizations, medical care, dental care, and necessities such as food and water [26]. The emotional and subsequent behavioural conditioning of the direct family members of drug abusers can be very significant. Parental inconsistency can result in a lack of structure and boundary setting for the child, which can result in inappropriate behavioural adjustment. Low parental expectation can result in adoption of the parents' paradigms and conforming to the parents' values and behaviour. The overriding negativism and increased neglect reported in households of abusers can result in the children being conditioned to create a crisis in order to gain attention. If emotionally deprived at home, resentment can lead miscarried expression of anger in other forms, which can impact both the home and the community. Ultimately such emotions can lead to self-medication with drugs and the potential to follow down the path of substance abuse [45].

### 3.2. Communities and Society

The cost to the community and society of drug abuse is colossal. Drug abuse has a significant impact on healthcare services, public services, and criminal justice system. Drugs that lead to intense intoxication such as alcohol, amphetamines, cocaine, and heroin are associated with huge costs in terms of damage to the user or the general public and property and loss of economic output. A large part of the health care budget is spent on treating drug users [46]. Alcohol alone is estimated to be involved in over half of all A&E department admissions [1]. Data published by the home office reports the estimated economic and social cost of Class A drugs alone to be around £15.4 billion per year [47]. Decreased social functioning related to drug abuse renders many dependant on state support with the home office estimating 80% of problem drug users claiming benefits at a cost of over £40 million a year [44]. Severe dependence and altered behaviour secondary to abuse are responsible for the most significant implications to communities and to society. Users requiring increasing amounts of drug to achieve the same high and to feed their on-going addiction often turn to crime to support their habit. Drug abusers are estimated to commit 36 million drug-motivated crimes each year which financially accounts for 90% of the total cost to society. 

## 4. Conclusion

Drug abuse has many damaging consequences not only for the individual, but also for the society as a whole. Underneath the pleura of social problems is a phenomenally complex mechanism involving tolerance, dependence, and addiction. Multiple brain areas are implicated in many functions such as reward, motivation, learning, inhibitory control, and executive function. Addictive drugs hijack the reward pathway that is intended for natural reinforcers and in doing so cause harm both acutely and chronically to individuals and society.

## Figures and Tables

**Table 1:** Acute effects of opioids and rebound withdrawal symptoms [12] commonly experienced upon cessation of heroin.

Acute action	Withdrawal sign
Analgesia	Pain and irritability
Respiratory depression	Hyperventilation
Euphoria	Dysphoria and depression
Relaxation and sleep	Restlessness and insomnia
Tranquilization	Fearfulness and hostility
Constipation	Diarrhoea
Decreased blood pressure	Increased blood pressure
Pupillary constriction	Pupillary dilation
Hypothermia	Hyperthermia
Reduced sex drive	Spontaneous ejaculation
Flushed warm skin	Cold skin
Drying of secretions	Lacrimation and runny nose

**Table 2:** Classification of illegal drugs [1].

	Class in misuse of drugs act	Comments
Ecstasy	A	Essentially 3,4-methylenedioxy-N-methylamphetamine (MDMA)
4-MTA	A	4-Methylthioamphetamine
LSD	A	Lysergic acid diethylamide
Cocaine	A	Includes crack cocaine
Heroin	A	Crude diamorphine
Street methadone	A	Diverted prescribed methadone
Amphetamine	B	—
Methylphenidate	B	For example, ritalin (methylphenidate)
Barbiturates	B	—
Buprenorphine	C	For example, temgesic, subutex
Benzodiazepines	C	For example, valium (diazepam), librium (chlordiazepoxide)
GHB	C	Gamma 4-hydroxybutyric acid
Anabolic steroids	C	—
Cannabis	C	—
Alcohol	—	Not controlled if over 18 years in UK
Alkyl nitrites	—	Not controlled
Ketamine	—	Not controlled at the time of assessment; controlled as class C since January 2007
Khat	—	Not controlled
Solvents	—	Not controlled; sales restricted
Tobacco	—	Not controlled if over 16 years in UK

## Appendix

For more details see Table 2.

## References

[1] Nutt D., King L. A., Saulsbury W., Blakemore C. (2007). Development of a rational scale to assess the harm of drugs of potential misuse. *The Lancet*.

[2] Trozak D. J., Gould W. M. (1984). Cocaine abuse and connective tissue disease. *Journal of the American Academy of Dermatology*.

[3] Westover A. N., McBride S., Haley R. W. (2007). Stroke in young adults who abuse amphetamines or cocaine: a population-based study of hospitalized patients. *Archives of General Psychiatry*.

[4] http://www.toxbase.org/.

[5] Carlson N. R. (1996). *Physiological Psychology*.

[6] Doll R., Peto R., Boreham J., Sutherland I. (2004). Mortality in relation to smoking: 50 Years' observations on male British doctors. *British Medical Journal*.

[7] Evidence-based guidelines for the pharmacological management of substance abuse, harmful use, addiction and comorbidity: recommendations from BAP.

[8] Health Protection Agency Shooting Up: infections among injecting drug users in the United Kingdom 2006.

[9] Frischer M., Goldberg D., Rahman M., Berney L. (1997). Mortality and survival among a cohort of drug injectors in Glasgow, 1982–1994. *Addiction*.

[10] Pagliaro L., Pagliaro A. M. (2004). Pagliaros comprehensive guide to drugs and substances of abuse. *The American Journal of Psychiatry*.

[11] Baigent M. F. (2003). Physical complications of substance abuse: what the psychiatrist needs to know. *Current Opinion in Psychiatry*.

[12] Fieldman R., Jerrold S., Quenzer L. (1997). *Principles of Neuropsychopharmocology*.

[13] Evins A. E., Green A. I., Kane J. M., Murray R. M. (2013). Does using marijuana increase the risk for developing schizophrenia?. *Journal of Clinical Psychiatry*.

[14] http://www.drugabuse.gov/drugs-abuse/commonly-abused-drugs/health-effects.

[15] Trozak D. J., Gould W. M. (1984). Cocaine abuse and connective tissue disease. *Journal of the American Academy of Dermatology*.

[16] Moore P. M., Richardson B. (1998). Neurology of the vasculitides and connective tissue diseases. *Journal of Neurology, Neurosurgery & Psychiatry*.

[17] Ramón P., Navascués R. A., Baltar J., Seco M., Alvarez J. (1999). Antiglomerular basement membrane antibody-mediated glomerulonephritis after intranasal cocaine use. *Nephron*.

[18] Westover A. N., McBride S., Haley R. W. (2007). Stroke in young adults who abuse amphetamines or cocaine: a population-based study of hospitalized patients. *Archives of General Psychiatry*.

[19] Vasica G., Tennant C. C. (2002). Cocaine use and cardiovascular complications. *Medical Journal of Australia*.

[20] National Institute on Alcohol Abuse and Alcoholism Brian centres involved in reward. http://pubs.niaaa.nih.gov/.

[21] Grilly D. (2006). *Drugs and Human Behaviour*.

[22] O'Brien C. P., Childress A. R., McLellan A. T., Ehrman R. (1993). Developing treatments that address classical conditioning. *NIDA Research Monograph Series*.

[23] Koob G. F. (1996). Drug addiction: the Yin and Yang of hedonic homeostasis. *Neuron*.

[24] Saah T. (2005). The evolutionary origins and significance of drug addiction. *Harm Reduction Journal*.

[25] Nesse R. M., Berridge K. C. (1997). Psychoactive drug use in evolutionary perspective. *Science*.

[26] King S. Substance abuse disorders: neurocircuitry and neurobiology. https://www.studentcentral.brighton.ac.uk/.

[27] Lubman D. I., Yücel M., Kettle J. W. (2009). Responsiveness to drug cues and natural rewards in opiate addiction: associations with later heroin use. *Archives of General Psychiatry*.

[28] Volkow N. D., Li T.-K. (2004). Drug addiction: the neurobiology of behaviour gone awry. *Nature Reviews Neuroscience*.

[29] King S. Reward and addictive behaviours. https://www.studentcentral.brighton.ac.uk/.

[30] Carlson N. (2005). *Foundations of Physiological Psychology*.

[31] Jorquez J. S. (1984). Heroin use in the Barrio: solving the problem of relapse or keeping the Tecato Gusano asleep. *American Journal of Drug and Alcohol Abuse*.

[32] Marsh B. (1961). The cycle of rehabilitation and relapse among heroin addicts. *Social Problems*.

[33] Stahl S. (2002). *Essential Psychopharmocology*.

[34] Franklin T. R., Acton P. D., Maldjian J. A. (2002). Decreased gray matter concentration in the insular, orbitofrontal, cingulate, and temporal cortices of cocaine patients. *Biological Psychiatry*.

[35] Fotros A., Casey K. F., Larcher K. (2013). Cocaine cue-induced dopamine release in amygdala and hippocampus: a high-resolution PET [^18^F] fallypride study in cocaine dependent participants. *Neuropsychopharmacology*.

[36] Borders T. F., Booth B. M., Falck R. S., Leukefeld C., Wang J., Carlson R. G. (2009). Longitudinal changes in drug use severity and physical health-related quality of life among untreated stimulant users. *Addictive Behaviors*.

[37] ACMD (2003). *Hidden Harm: Responding to the Needs of Children of Problem Drug Users*.

[38] Kuczkowski K. M. (2002). Cocaine abuse in pregnancy—anesthetic implications. *International Journal of Obstetric Anesthesia*.

[39] The National Survey on Drug Use and Health. https://nsduhweb.rti.org/.

[40] Bateman D. A., Chiriboga C. A. (2000). Dose-response effect of cocaine on newborn head circumference. *Pediatrics*.

[41] PhyOrg.com Prenatal cocaine exposure impairs infants' response to stress. http://phys.org/news151690493.html.

[42] Ackerman J. P., Riggins T., Black M. M. (2010). A review of the effects of prenatal cocaine exposure among school-aged children. *Pediatrics*.

[43] Pinel J. (2006). *Biopsychology*.

[44] National Drug Intelligence Center http://www.justice.gov/archive/ndic/.

[45] Reilly D. M., Kaufman E., Kaufmann P. (1992). Drug-abusing families: intrafamilial dynamics and brief triphasic treatment. *Family Therapy of Drug and Alcohol Abuse*.

[46] Pinel J. (2006). *Biopsychology*.

[47] Gordon L., Tinsley L., Godfrey C., Parrott S. (2006). The economic and social costs of class A drug use in England and Wales, 2003/04 (Measuring different aspects of problem drug use: methodological developments). *Home Office Online Report*.

